# Isolation and Characterization of φkm18p, a Novel Lytic Phage with Therapeutic Potential against Extensively Drug Resistant *Acinetobacter baumannii*

**DOI:** 10.1371/journal.pone.0046537

**Published:** 2012-10-05

**Authors:** Gwan-Han Shen, Jiun-Ling Wang, Fu-Shyan Wen, Kai-Ming Chang, Chih-Feng Kuo, Chun-Hung Lin, Huei-Ru Luo, Chih-Hsin Hung

**Affiliations:** 1 Division of Respiratory and Critical Care Medicine, Department of Internal Medicine, Taichung Veterans General Hospital, Taichung, Taiwan, ROC; 2 Department of Respiratory Therapy, College of Health Care, China Medical University, Taichung, Taiwan, ROC; 3 School of Chinese Medicine for Post Baccalaureate, I-Shou University, Kaohsiung County, Taiwan, ROC; 4 Department of Internal Medicine, E-Da Hospital, Kaohsiung County, Taiwan; 5 Department of Life Science, National Chung Hsing University, Taichung, Taiwan, ROC; 6 Department of Nursing, I-Shou University, Kaoshiung, Taiwan, ROC; 7 Department of Chemical Engineering, and Institute of Biotechnology and Chemical Engineering, I-Shou University, Kaoshiung, Taiwan, ROC; Los Angeles Biomedical Research Institute, United States of America

## Abstract

**Aims:**

To isolate phages against extensively drug resistant *Acinetobacter baumannii* (XDRAB) and characterize the highest lytic capability phage as a model to evaluate the potential on phage therapy.

**Methods and Results:**

Eight phages were isolated from hospital sewage and showed narrow host spectrum. Phage φkm18p was able to effectively lyse the most XDRAB. It has a dsDNA genome of 45 kb in size and hexagonal head of about 59 nm in diameter and no tail. Bacterial population decreased quickly from 10^8^ CFU ml^−1^ to 10^3^ CFU ml^−1^ in 30 min by φkm18p. The 185 kDa lysis protein encoded by φkm18p genome was detected when the extracted protein did not boil before SDS-PAGE; it showed that the lysis protein is a complex rather than a monomer. Phage φkm18p improved human lung epithelial cells survival rates when they were incubated with *A. baumannii*. Combination of phages (φkm18p, φTZ1 and φ314) as a cocktail could lyse all genotype-varying XDRAB isolates.

**Conclusion:**

Infections with XDRAB are extremely difficult to treat and development of a phage cocktails therapy could be a therapeutic alternative in the future. Phage φkm18p is a good candidate for inclusion in phage cocktails.

## Introduction

*Acinetobacter baumannii*, a gram-negative, non-fermentative bacterium, is one of the most important nosocomial pathogens in hospitals, especially in intensive care units. *A. baumannii* is an opportunistic pathogen that causes serious nosocomial outbreaks. The mortality rate is elevated and the length of hospitalization is prolonged if drug resistance develops [1], [2]. In recent years, multi-drug resistant *A. baumannii* have been spread worldwide including Taiwan [3]–[5]. More recently, the term “extensively drug resistant” *A. baumannii* (XDRAB) has been used to characterize the bacterial isolates resistant to all authorized antibiotics except 2 category of antibiotic e.g. tigecycline and polymyxins [4], [6], [7]. Given the increasing prevalence of XDRAB infection with high mortality rate, new antibiotics and are needed to combat this challenge, and one possible solution is the therapeutic use of phages [8]–[13].

Phages have been used as pharmaceutical agents for more than 90 years. Because of the threat of serious multi-drug resistant bacterium infection, there has been renewed interest in the use of phages for the treatment of infectious diseases of humans and other animals [8]–[13]. Numerous animal studies have demonstrated the effectiveness of phages against multi-drug resistant bacteria, such as vancomycin-resistant *Enterococcus faecium* [14], *Escherichia coli* [15], *Pseudomonas aeruginosa* [16]–[17], *Staphylococcus. aureus* [18] and *Vibrio vulnificus* [19]. Some phages of common bacterial pathogens, such as *Listeria monocytogenes*, *Salmonella* spp. and *Pseudomonas*, have been approved for commercial use in food preservation [20]–[21]

The phages of *Acinetobacter* spp. have been studied previously, but most were used for transduction, phage typing or classification of phages [22]–[25]. Previous reports have described the phage BS46, which specifically infects *A. baumannii* AC54 and protects infected mice from death [26]. An ssRNA phage, AP205, that propagates in *Acinetobacter* genospecies 16 (*A. radioresistens*) was also isolated and analyzed [27]. Due to the limited efficacy and potential side effect of current antibiotic against XDR-AB, we want to isolate phages against XDR-AB and characterize highest lytic capability phage as a model to evaluate the potential on phage therapy.

## Materials and Methods

### Bacterial strains

Thirty-four clinical strains of *A. baumannii* from five medical centers (Taichung Veterans General Hospital, National Taiwan University Hospital, Kaohsiung Medical University Chung-Ho Memorial Hospital, Hualien Tzu Chi Medical Center and Tri-Service General Hospital) were used in this study (Table 1). All isolates underwent MIC testing for various drugs, including carbapenems, anti-pseudomonal cephalosporins, anti-pseudomonal penicillins, monobactams, aminoglycosides, tetracyclines, fluoroquinolones, sulbactam and polymyxins, using CLSI (Clinical and Laboratory Standards Institute) guidelines (National Committee for Clinical Laboratory Standards, USA).

**Table 1:** The antibiotic sensitivity results of reference strains and the spot test of eight phages against the reference strains of *A. baumannii* used in this study.

Strain	Phages								Antibiotics sensitivity†	Source‡
	φkm18p	φTZ1	φ2449N	φAb21	φKM5	φ314	φ134	φ2449p		
Ab016	+++*	−	+	−	−	−	−	−	CS	TVG
Ab019	+++	−	−	−	−	−	−	+++	CS	TVG
Ab14	−*	−	+	−	−	−	+++	+	CS	TVG
Ab15	−	−	+++	−	−	+++	−	+++	CS	TVG
Ab16	−	−	+	−	−	+++	−	+++	CS	TVG
AB19	−	+++	−	−	−	−	−	−	CS	TVG
Ab23	+++	−	−	−	−	−	+++	−	CS	TVG
Ab26	−	−	+	−	−	+++	−	+++	CS	TVG
Ab29	+++	−	−	−	−	−	+++	+++	CS	TVG
Ab35	+++	−	−	−	−	−	+++	+++	CS	TVG
Ab39	−	−	−	−	−	+++	−	+	CS	TVG
Ab40	+++	−	−	−	−	−	+++	+	CS	TVG
Ab54	−	−	−	−	−	−	−	+++	CS	TVG
TVG55	−	−	+++	−	−	+++	−	−	CRAB	TVG
NTU2449	−	−	+++	−	−	+	−	−	CRAB	NTU
KM5	−	−	−	−	+++	−	−	−	MDRAB	KM
Ab21	+++	−	−	+++	−	−	−	−	MDRAB	TVG
TVG68	+++	−	−	−	−	−	+++	−	MDRAB	TVG
Ab002	+++	−	+	−	−	−	+++	−	XDR-AB	TVG
Ab010	+++	−	−	−	−	−	+++	+	XDR-AB	TVG
Ab011	+++	−	−	−	−	−	+++	−	XDR-AB	TVG
Ab015	+++	−	−	−	−	−	+++	+	XDR-AB	TVG
Ab021	+++	−	−	−	−	−	+++	+	XDR-AB	TVG
KM16	−	+++	−	−	−	−	−	−	XDR-AB	KM
KM18	+++	−	−	−	−	−	+++	+	XDR-AB	KM
TSG2	−	−	+	−	−	+++	−	−	XDR-AB	TSG
TSG4	−	−	+++	−	−	+++	−	−	XDR-AB	TSG
TSG5	−	+++	−	−	−	−	−	−	XDR-AB	TSG
TSG6	−	+++	−	−	−	−	−	−	XDR-AB	TSG
TVG46	+++	−	−	−	−	−	+++	−	XDR-AB	TVG
TVG52	−		+++	−	−	+++	−	−	XDR-AB	TVG
TVG57	−	+++	−	−	−	−	−	−	XDR-AB	TVG
TVG58	−	+++	−	−	−	−	−	−	XDR-AB	TVG
TZ1	−	+++	−	−	−	−	−	−	XDR-AB	TZ

* “−”: no effect; “+”: turbid zone; “+++”: clear zone.

† CS: carbapenem sensitive *A. baumannii*; CRAB: carbapenem resistant *A. baumannii* (including imipenem and meropenem resistant); MDRAB: multi-drug resistant (resistance to 3 or more of the following classes of antipseudomonal cephalosporin, antipseudomonal carbapenem, ampicillin-sulbactam, fluoroquinolone, aminoglycoside); XDR-AB: extensively drug resistant *Acinetobacter baumannii* (resistant to all antibiotics except colistin or tigecycline).

‡ TVG: Taichung Veterans General Hospital, Medical Center; NTU: National Taiwan University Hospital, Medical Center; KM: Kaohsiung Medical University, Chung-Ho Memorial Hospital; TZ: Hualien Tzu Chi Medical Center.; TSG: Tri-Service General Hospital, Medical Center.

### Phage isolation

Sewage samples were collected from various aquatic ecosystems in the Taichung Veterans General Hospital (Taichung, Taiwan) and E-Da Hospital (Kaoshiung, Taiwan) for phage isolation. The presence of phages was investigated using a phage enrichment technique [28]. A 50-ml supernatant form centrifugated sewage was mixed with 50 ml double-strength Luria-Bertani broth containing exponential-phase *A. baumannii* strains. After a 24-h incubation at 37°C, the bacterial cells were centrifuged and the supernatant was filtered through a 0.22- µm membrane filter. Then, 10 µl of filtrate and the indicator strain were mixed with soft agar and poured onto an LB agar plate. Phage plaques would be visible after overnight incubation at 37°C. Single plaques were selected by a sterile tooth pick and suspended in 500 µl PBS. Followed by double layer plaque assay was perform to obtain pure phage strains.

### Purification of phage particles

Phage lysate (ca. 1×10^10^ PFU ml^−1^) was centrifuged at 14,700×g for 30 min at 4°C, and then the supernatant was filtered through a 0.22- µm filter. The filtered supernatant was treated with nucleases (0.25 mg each of DNase and RNase per ml) for 1 h at 37°C to digest the bacterial genomic DNA. Polyethylene glycol (average molecular weight, 8000 Dalton) and NaCl were added to the filtered phage suspension to yield final concentrations of 3% and 0.33 M, respectively, and the mixture was stored at 4°C for 2 h. The phage in the mixture were collected by centrifugation at 14,700×g for 30 min at 4°C, and the pellet was resuspended in PBS buffer. This condensed phage suspension was loaded onto a discontinuous CsCl gradient diluted in PBS buffer and centrifuged at 35,000 rpm for 9 h at 4°C in a Beckman L-90 ultracentrifuge with a SW41Ti rotor. The banded phage particles were collected and dialyzed against PBS buffer (1 h) three times. The purified phage suspension was stored at 4°C until use.

### In vitro test of optimal phage titer to challenge bacterium

To determine the optimal titer for phage to decrease the bacterial concentration was according to the previously described [29]. An overnight host culture was transferred to 30 ml fresh LB broth medium (OD_600_ = 0.1) and grown at 37°C until the concentration reached OD_600_ = 1.0. Phage φkm18p at different multiplicity of infection (MOI of 0.01, 0.1, 1 and 10) were inoculated to the fresh culture (OD_600_ = 1.0) separately and incubated at 37°C. The phage-infected culture sample was removed 1 ml at interval time and was centrifuged at 12,000 for 5 min to separate the supernatant and bacterium pellet. Then the pellet was washed with phosphate-buffer saline (PBS) one time, and resuspended with 1 ml PBS. The suspension of pellet was serial diluted and spread on the LB agar (1.5%) plate to determine the visible bacteria.

### Isolation and restriction enzyme digestion of phage DNA

Phage DNA was extracted from the purified phage particles (1.0×10^10^ PFU ml^−1^) following the procedure described in the Viral Nucleic Extraction Kit II (Geneaid, Taipei, Taiwan). DNA samples were digested with the restriction enzyme *Hin*cII according to the supplier's instructions (New England Biolabs, Beverly, Mass). The digested DNA was analyzed by electrophoresis through a 0.8% agarose gel prepared with 0.5× Tris-Borate-EDTA (TBE) running buffer.

### Pulsed Field Gel Electrophoresis (PFGE)

Pulsed-field gel electrophoresis was performed according to a modification of the protocol described by Tseng *et al.* [30]. Purified phage suspension (ca. 1×10^10^ PFU ml^−1^) was mixed with an equal volume of molten 1% agarose (SeaKem Gold agarose, Cambrex), which was allowed to solidify in a mold (15-mm length×1-mm width×10-mm depth). The solid block was lysed at 55°C overnight by proteinase K (10 mg ml^−1^) in cell lysis buffer (50 mmol l^−1^, Tris, 50 mmol l^−1^ EDTA at pH 8.0, 1% Sarcosine). After the proteinase K lysis step, the block of agarose was washed in TE buffer three times for 30 min each. For restriction enzyme digestion, a plug (15-mm×1-mm×2-mm) was cut from the agarose block containing the phage genomic DNA and washed in sterile distilled water three times. The phage DNA was subjected to restriction digest analysis according to the manufacturer's instructions. The plugs were loaded onto 1% agarose prepared with 0.5× TBE (pH 8.0) running buffer. The restriction fragments were separated by PFGE (using the CHEF-DR III apparatus from Bio-Rad, Richmond, CA, USA) at 14°C with a ramping time of 2 s to 12 s for 8 h, at a field strength of 6 V/cm.

### Electron microscopic examination of phage morphology

Samples for electron microscopic examination were prepared as follows: equal amounts of phage suspension (10^10^ PFU ml^−1^) and 0.1% bacitracin were mixed well, and one drop of this mixture was spotted onto a mesh grid for 3 min. The edge of the grid was touched with a piece of Whatman filter paper to drain away any excess suspension and the grid was then stained with 2% uranyl acetate for 30 s. The prepared samples were examined under a JEM-2000 EX II electron microscope (JEOL, Japan) at an operating voltage of 80 kV.

### SDS-PAGE under non-denaturing conditions

The method was according to the description by Jyothistri *et al.* [31]. To investigate the lysis protein of phage φkm18p, phage lysate was centrifuged at 14,700×g for 30 min at 4°C, and the supernatant was filtered through a 0.22- µm filter. Ammonium sulfate (30%) was added to precipitate the proteins in the filtered suspension, and then the suspension was dialyzed with PBS buffer (137 mmol l^−1^ NaCl, 2.7 mmol l^−1^ KCl, 10 mmol l^−1^ Na_2_HPO_4_, 2 mmol l^−1^ KH_2_PO_4_). The protein suspension was mixed with sample buffer without β-mercaptoethanol. The samples were then run on SDS-PAGE without boiling. The resolved gel containing the extracted proteins was plated onto L-agar and soft agar mixed with *A. baumannii* KM18 was poured onto the gel, and incubated at 37°C overnight. Clear zones on the overlay indicate lytic proteins.

### A549 cell survival test

To ensure the safety of phage therapy, we investigated the toxicity of crude isolated phages to A549 human lung epithelial cells. A549 cells (10^5^ cell well^−1^) were plated in a 24-well ELISA plate and incubated for 12 h (37°C, 5% CO_2_) in 100 µl per well of Dulbecco's modified Eagle medium (DMEM) (Sigma, D5523, USA) supplemented with 5% fetal calf serum. Then, 10^6^ CFU *A. baumannii* KM18 were added to the cells, followed by the immediate addition of phage at different titers (MOI = 0, 1000, 1, 0.1, 0.01). The cells were cultured for 24 h at 37°C in a 5% CO_2_ incubator. As a control, A549 cells were treated with 10^9^ PFU phages without *A. baumannii* KM18. After incubation, the wells were washed twice with PBS and incubated with trypsin-EDTA solution (0.05% trypsin, 0.5 mM EDTA-tetra sodium) to allow surviving cells to separate from the well. The cell counts of surviving A549 cells suspended in trypsin solution were determined by microscopic observation (Nikon, E2000, Japan).

## Results

### Antibiotic susceptibility and bacterial strain identification

Table 1 showed that 16 strains of *A. baumannii* were XDR-AB (resistant to all antibiotics except colistin or tigecycline). Three strains were carbapenem-resistant (including imipenem- and meropenem-resistant). Two strains resistant to fluoroquinolone were termed MDR-AB. The genomic DNA of these 34 strains was digested with *Apa*I and separated by PFGE. Different DNA patterns were seen after PFGE and indicated that all 34 bacterium were different strains (data not shown).

### Isolation of eight lytic phages

Thirty-four clinical *A. baumannii* strains were used as host indicators for the isolation of lytic phages. Finally, eight lytic phages were isolated and their host ranges were determined by spot tests on these host strains and presented in Table 1. Phages φkm18p and φ2449p were isolated from the sewage of Taichung Veterans General Hospital, and the other phages, φTZ1, φ2449N, φAb21, φKM5, φ314 and φ134, were isolated from the sewage of E-Da Hospital. Table 1 shows that 15 of 34 indicated strains were sensitive to φkm18p and distributed in the carbapenem-sensitive and XDRAB groups. Phage φ134 was found to have almost the same host range as φkm18p. Phage φ2449p was most virulent to carbapenem-sensitive strains, but was non-lytic to XDRAB strains. Only three bacterial strains were subject to lysogeny by φ2449p. The hosts of φTZ1 and φ314 did not overlap with those of phage φkm18p. Therefore, this combination of phages could be used as a cocktail to target all XDRAB. Phages φAb21 and φKM5 presented the most narrow host range as only one bacterium strain was sensitive to each of them. From the results shown in Table 1, phage φkm18p was found to be the most powerful phage. The spot tests of phage φkm18p on clinical strains of *A. baumannii* showed the widest range of hosts, including imipenem-susceptible and XDRAB strains. Compared with other phages, φkm18p produced more lysis of XDRAB strains than other phages did. We also found that the plaques of φkm18p were 2 mm larger in diameter than those of the other seven phages and that φkm18p lysed the bacteria more quickly in liquid medium (data not shown). Because most XDRAB strains were sensitive to φkm18p and given that φkm18p could lyse most strains in this study, it was chosen for further study. In addition to φkm18p, the remaining phages were kept as stocks for a phage bank. In the future, their therapeutic efficacy in different mixtures could be evaluated.

### Phage φkm18p lysed *A. baumannii* KM18 in 30 minutes

**Figure 1:**
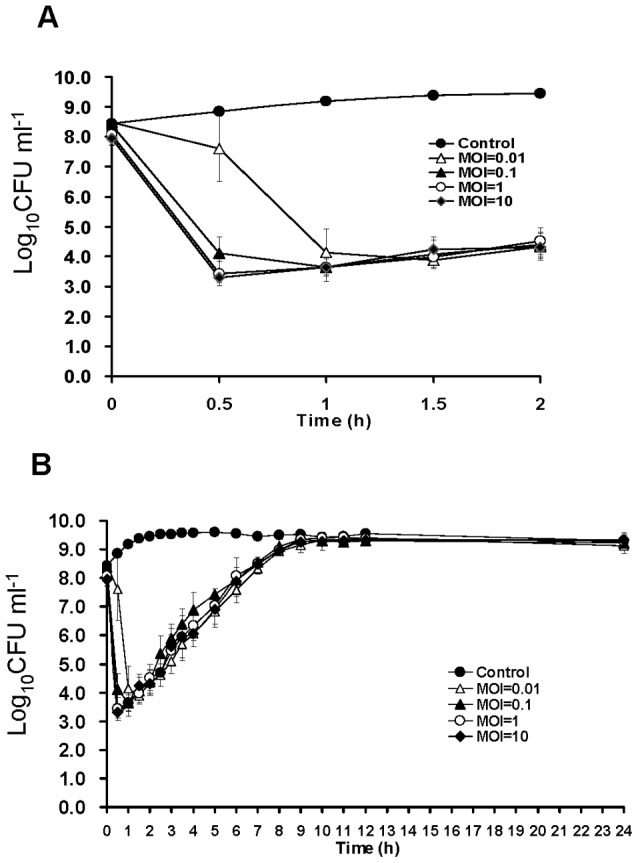
Relationship between phage titers and inhibition efficiency. Phages φkm18p were used at different titers to infect host cells in order to determine the best titer for lysis of the host during 2 hr (A) and 24 h (B) periods. Phage concentration: (•) no phage added, (○) MOI = 1, (▾) MOI = 0.1 and (▵) MOI = 0.01.

Phage φkm18p lysed *A. baumannii* KM18 quickly as indicated by big, clear plaques. The lytic activity of φkm18p was examined by inoculating phages into *Ac. baumannii* KM18 (5×10^8^ CFU ml^−1^). As shown in Fig. 1 (A), the phage titers with MOI of 10, 1, 0.1 and 0.01 decreased KM18 from 10^8^ CFU ml^−1^ to 10^3^ CFU ml^−1^ in 1.5 h. From detailed analysis, an MOI of 1 reduced *A. baumannii* KM18 to the residual titer of 10^3^ CFU ml^−1^ quickly in 30 min, but MOIs of 0.1 and 0.01 were also sufficient to reduce *A. baumannii* KM18 to the same point in 1 h and 1.5 h, respectively. The lytic activity of an MOI of 10 was also tested; it showed the same results as for an MOI of 1. A higher dose of phage was better for reducing the bacterium concentration but is not absolutely necessary for lysis. The bacterium concentration was raised after phage inoculating 2 h later, and finally all the phage-infected bacterium concentrations were raised as the control after the phage was inoculated 10 h later (Fig. 1 (B)).

### Electron micrograph of phage morphology

**Figure 2:**
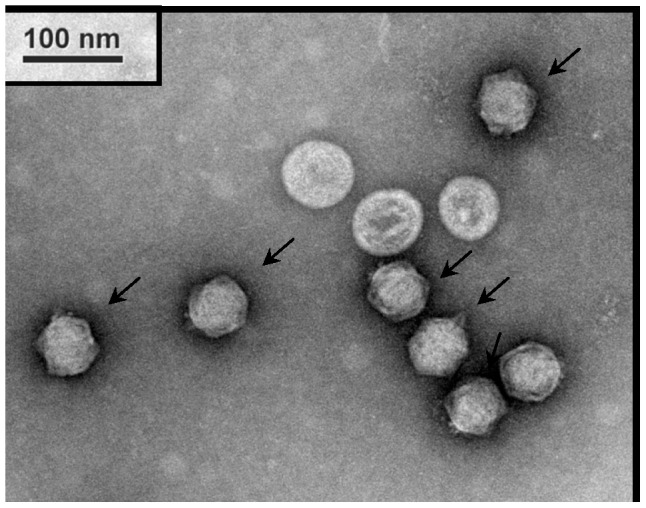
Electron micrographs of *A. baumannii* phage φkm18p. Phage suspension was loaded onto a copper grid, stained with 2% uranyl acetate and observed with transmission electron microscopy. The phage particles (the arrows indicated) showed a hexagonal head about 59 nm in diameter that lacks a neck and tail.

An electron micrograph of phage particles revealed that the phage has a hexagonal head 59 nm in diameter and lacks a tail (Fig. 2). The φkm18p phage particles were concentrated in a visible band at a density of 1.5 g ml^−1^ in a CsCl gradient. Phage φkm18p was similar in morphology and size to phage PM2 [32] and should be assigned to the family *Corticoviridae* according to the taxonomic database of ICTVdB [33].

### Phage genome size and protein profiles

**Figure 3:**
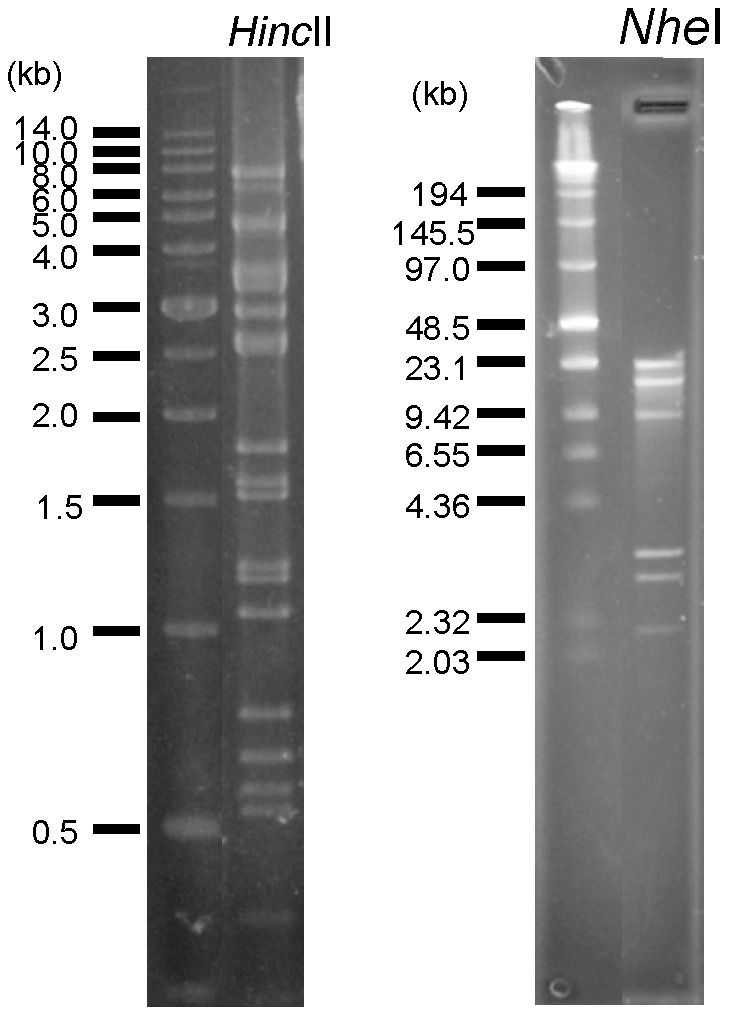
Phage φkm18p genomic DNA restriction patterns and size determination. (A) Agarose gel electrophoresis of *Hinc*II fragments. (B) *Nhe*I fragments separated by PFGE. The total size of genomic DNA was estimated at approximately 45 kb.

**Figure 4:**
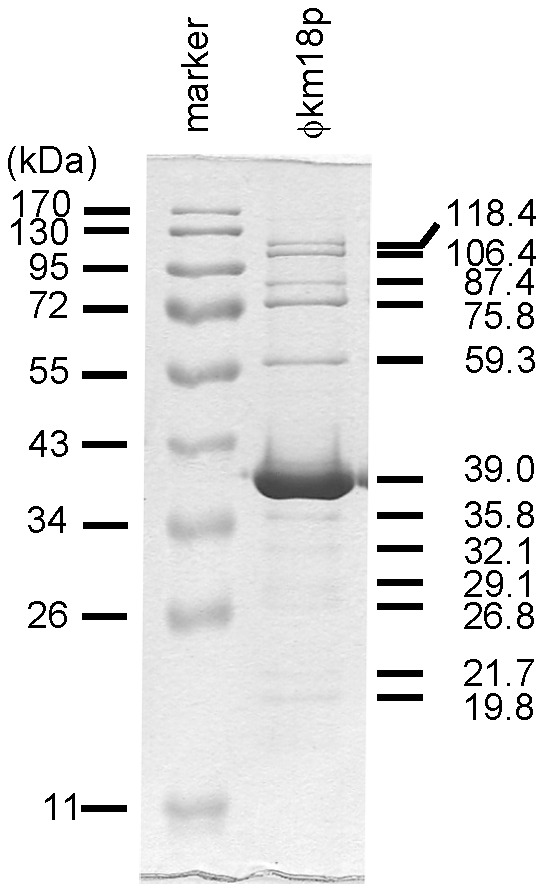
The phage particle virion proteins were separated by SDS-PAGE. The phage particles (1.5×10^10^ PFU) were boiled with cracking buffer and subjected to SDS-PAGE using 12% gels. The most abundant protein was 39 kDa.

Restriction fragments of phage DNA were separated by gel electrophoresis and PFGE to determine the genome size. *Hinc*II fragments of phage DNA were separated by gel electrophoresis, resulting in DNA patterns that included double bands for approximately 19 fragments (Fig. 3, (A)). The optimal second enzyme *Nhe*I was also used to digest genomic DNA, resulting in six fragments visible on a PFGE gel (Fig. 3, (B)). From the *Hinc*II and *Nhe*I digest patterns, the genome size of phage φkm18p was found to be approximately 45 kb. Phage virion structural proteins were at least 12 protein patterns and the most abundant protein was 39 kDa which was predicted to be a major coat protein (Fig. 4).

### Phage endolysin activity was shown by bacterium overlay of a PAGE gel

**Figure 5:**
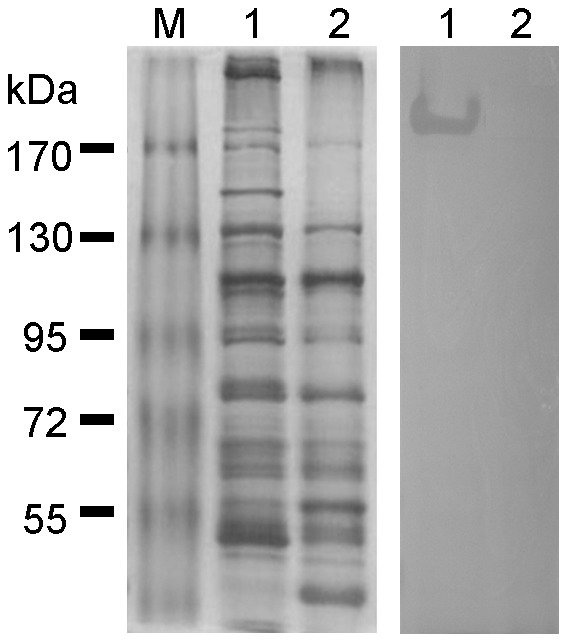
Phage endolysin activity and size were determined by an overlay assay. The protein suspension extract (about 13 mg) from the phage lysate was mixed with sample buffer without β-mercaptoethanol. Unboiled samples were analyzed by SDS-PAGE. The control set used a sample buffer containing β-mercaptoethanol and the sample was boiled for 10 min. The SDS-PAGE gel was poured onto a plate and soft agar mixed with *Ac. baumannii* KM18 was overlaid. The protein with endolysin activity produced a clear region on the overlay.

Upon release, the phages burst the bacteria, causing endolysin to flow outside into the medium. Proteins of supernatant were precipitated by 30% ammonia sulfate and separated by SDS-PAGE, as described in the materials and methods. Bacterium overlay on SDS-PAGE showed a clear band at 185 kDa, but clear band disappeared when the sample was boiling treatment before electrophoresis (Fig. 5). From this result, we suggest that the endolysin functions in a complex rather than as a monomer.

### Phage ensures A549 cell survival under bacterial infection

**Figure 6:**
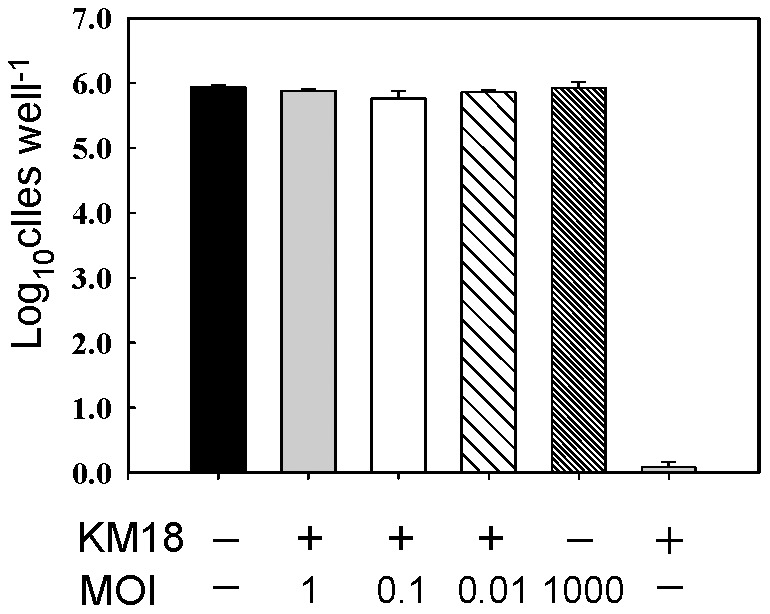
Protection efficacy test of φkm18p on A549 human lung epithelial cells. A549 cells were distributed in a 24-well ELISA plate (10^5^ cells well^−1^) and incubated for 12 h. The cells were infected with 10^6^ CFU *Ac. baumannii* KM18. Phage at different titers (MOI = 0, 1000, 1, 0.1, 0.01) was added to the cells. The “+” indicates the addition of phage and “−” means phage were not added. The cell counts of surviving A549 cells suspended in trypsin solution were determined by examination under the microscope (Nikon, E2000, Japan). Results were confirmed by repeating the experiments three times.

To ensure the safety of phage therapy, A549 human lung epithelial cells were used as an indicator for phage toxicity. The phage φkm18p was found to provide the highest protection against *A. baumannii* KM18 infection of cells (10^5^ cells) (Fig. 6). Phage at an MOI of 1, 0.1 or 0.01 enabled cells inoculated with *A. baumannii* KM18 (10^6^ CFU) to survive as well as uninoculated controls. Cells treated with phages at an MOI of 1000 (10^9^ PFU), but not inoculated with bacteria, survived as well as the control cells. Cells inoculated with the bacterial strain *A. baumannii* KM18 only without any phage treatment were completely killed. The results showed that φkm18p eliminated bacteria and protected A549 cells from immediate killing by KM18 bacteria. They also suggested that a high dose of phages did not impact A549 survival.

## Discussion

Phage φkm18p has assigned to the family *Corticoviridae* and was a new phage of *Ac. baumannii*. Previously, several phage species have been isolated. They belong to the *Myoviridae*, *Siphoviridae* and *Podoviridae* families of tailed phages [23]. An ssRNA bacteriophage of *Acinetobacter* from the family *Leviviridae* was also identified [27].

Our purpose was to investigate the possibility of phage therapy to eliminate XDRAB and found phage φkm18p was a good candidate. From the report of Soothill [26], the phage BS46 provided protection to *A. baumannii-*infected mice, indicating that it is possible to control antibiotic-resistant *A. baumannii* by phage therapy. In our study, clinical *A. baumannii* isolates were used to survey lytic phages. We chose the most active phages for further analysis of their plaque morphology, effect on bacterial growth, genome size, endolysin activity and protection of cells. We examined the antibiotic sensitivity of these 34 strains of *A. baumannii*, and classified them as CS, CRAB, MDRAB and XDRAB. All of the bacterial strains were sensitive to the phages isolated in this study. The phage φkm18p was unique among the phage isolates; 15 bacterial strains were sensitive to it, including seven XDRAB strains. Although host specificity of phages is a major limitation for therapy, a phage cocktail could combat bacteria in an emergency. Table 1 shows that the host ranges of the studied phages are narrow. Phage φkm18p (44.1%) had the broadest range of hosts and triggered lysis in all hosts; the other phages showed decreased efficiency of infecting these strains. The major drawback of phages is their narrow host range; therefore, a large combination of phages is needed for therapy. The lytic and lysogeny phage, such as φ2449p was most virulent to carbapenem-sensitive strains, but non-lytic and lyogeny to XDRAB strains was not a suitable candidate for cocktail combination. The lytic or lysogeny pathway was determined when the phage DNA was injected into host, if the genome of phage integrate into host DNA and coexist in it, some antibiotic resistant gene or other host fragment would be assembly into the new synthesis phages. Using the lysogenic phage as the agent would dangerously transfer the multidrug resistant genes or toxic genes to other hosts. The candidates of phage for biocontrol or phage therapy should be obligate lytic to bacterium. These phages shouldn't have integrases; would degrade the host genome DNA and lack the opportunities to coexist with their host. However, many genes of isolated phages still lack function information and database matches [34]. We tried to isolate phages to combat all of the bacterial strains shown in Table 1. None of the phages had efficient activity against most bacterial strains, but each bacterial strain could be lysed by at least one phage. We believe that every derived bacterial strain can be lysed by one type of phage in nature.

Phage φkm18 decreased the concentration of *A. baumannii* from 10^9^ CFU ml^−1^ to 10^3^ CFU ml^−1^ within 30 min and maintained this concentration for about 2 h. But the high titer of phage with MOI = 10 presented the same lytic activity as MOI = 1 (Fig. 1 (A)). It was thought that the higher titer of phage inoculation would get higher efficiency on cleaning the bacterium, but the complex dynamic interactions between hosts and the phages always have to optimize the efficiency of phage as the agent for phage therapy. Each phage has its' optimal concentration to interact to its' host. Higher titer of phage would raise the attaching rate on the host and decrease the bacterium concentration quickly. But sometimes phage would induce the phage-immune system and refuses other phage infection. There is also possible that the phage titer is saturate and receptors for phage are occupied and the bacterium reduction will not increase with increase MOI values. [35]–[36]. Thus it would be a property of phage that only interacts with a small portion of hosts in the purpose to their reproductivity. The burst size would be the factor to explain the phages could evolve to utilize a small proportion of host, probability of death of hosts and the time for phage production. But also interpret that the maturation of progeny is asynchronous with actual time of lysis and leaving the flexible control point [37]–[38]. As with any phage-host interaction, bacterial growth increases over time [29], [39]–[40]. Another possible explanation for this finding is the production of mutant phage-resistant strains. Phage-resistant bacteria have been mentioned in many articles [41]–[43]. However, phage sensitivities also resulted in a change in Omp (outer membrane protein) and LPS (lipopolysaccharide) profiles of the bacteria [42]. Bacterial growth eventually rises after infection by one type of phage, but a cocktail of lytic phages completely eliminates the pathogen [29].

Human lung epithelial cells A549 (10^5^ cells well^−1^) were killed when inoculated with 10^4^ CFU of *A. baumannii* KM18. In this study, a higher concentration (10^6^ CFU) of bacteria was used to infect the cells and to investigate the protective efficacy of phages on the cells. The data presented in Fig. 6 showed that φkm18p increased the survival rate after 24-h incubation to the same rate as the control without added pathogen. Otherwise, the phage φkm18p did not affect the growth of A549 cells, indicating that the phage φkm18p is a potential candidate for phage therapy. Also, bacteria infected with phage φkm18p started to proliferate at two hour after infection, causing the growth curve to rise again. The bacterial density was also found to increase after a 24-h incubation of A549 cells, *A. baumannii* KM18 and φkm18p (Fig. 1(B)). But why did the bacterial cells survive under a high titer of *A. baumannii* KM18? We analyzed the surviving bacteria and found that phage-resistant bacterial strains had developed (data not shown). Further animal model experiments should be perform to confirm that phage φkm18p protect animal infected by XDRAB.

When the phage particles matured in the bacteria, the endolysin encoded by phage genes hydrolyzed the cell wall of the host and disrupted the bacterium, causing the phage particles to burst [18], [44]. When the bacteria were destroyed, endolysin was also released into the lysate. The protein with lytic activity showed a clear pattern on the bacterial overlay, indicating a protein size of about 185 kDa (Fig. 5). From the results, it was suggested that the lytic protein is a multimer because the protein with lytic activity disappeared after denaturation by boiling. Purified phage-encoded peptidoglycan hydrolase (lysin) is also reported to be effective for the treatment of bacterial infections [43].

Current treatment of XDRAB included tigecycline and colistin [45]–[46]. Colistin is limited by its potential renal toxicity [45]. Tigecycline is limited by lack of efficacy bloodstream and urinary tract infection [46]. Besides, emerging resistance to colistin and tigecycline has been reported [3], [4], [7]. Phage therapy is a revitalized therapy against bacterial infectious diseases. Previous reports have shown that appropriate administration of living phages can be used to manage life-threatening infectious diseases caused by gram-negative bacteria, such as *E. coli*, *A. baumannii*, *K. pneumoniae* and *P. aeruginosa* [26], [47]–[48]. The Poles and the Soviets have administered phages orally, topically and systemically to treat different antibiotic-resistant pathogens, including *Staphylococcus*, *Streptococcus*, *Klebsiella*, *Escherichia*, *Proteus*, *Pseudomonas*, *Shigella*, *Salmonella* and *Acinetobacter* spp. [8]. The phages specific for XDRAB described in this study will hopefully be prepared to cope with the resistance.
